# Unrecognized Atrial Septal Defect Presenting with Postoperative Occipital Infarction

**DOI:** 10.3390/diagnostics16060907

**Published:** 2026-03-19

**Authors:** Jasper Lin, Vinicius Carraro do Nascimento, Jeremy Hefford, Tony Vo, Maria Gabriela Matta

**Affiliations:** 1 Department of Cardiology, Division of Specialist Medical Services, Gold Coast Hospital and Health Services, Southport, QLD 4215, Australia; 2 Department of Interventional Neuroradiology, Gold Coast University Hospital, Southport, QLD 4215, Australia

**Keywords:** atrial septal defect, paradoxical embolism, embolic stroke, percutaneous closure

## Abstract

A 49-year-old woman developed acute homonymous hemianopia three days after elective surgery. Neuroimaging confirmed an embolic right occipital infarction. Transthoracic echocardiography demonstrated early bubble passage, and transesophageal imaging identified a 4.6 mm ostium secundum atrial septal defect. The defect was successfully closed percutaneously with complete shunt resolution. This case emphasizes the value of targeted multimodality imaging in uncovering occult interatrial shunts and highlights presumed paradoxical embolism as a potential mechanism of postoperative or cryptogenic stroke.

**Figure 1:**
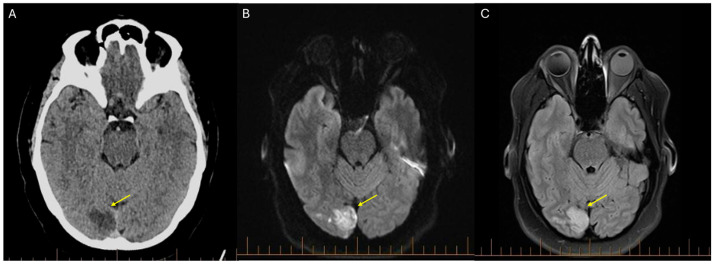
**Multimodality neuroimaging demonstrating acute right occipital infarction (yellow arrows).** A 49-year-old woman presented to the Emergency Department with blurred vision three days after an elective total laparoscopic hysterectomy for uterine fibroids. The procedure lasted approximately two hours, and pharmacological venous thromboembolism prophylaxis was not administered perioperatively according to the surgical perioperative protocol. Her medical history included uterine fibroids and seropositive rheumatoid arthritis treated with methotrexate (25 mg weekly) and etanercept (50 mg weekly), and she was an active smoker. Electrocardiography showed normal sinus rhythm. Neurological examination demonstrated left homonymous hemianopia, reflecting loss of the left visual hemifield in both eyes. Given a symptom onset of 72 h, a subacute cerebrovascular accident was suspected, and a code stroke was activated, despite a National Institutes of Health Stroke Scale (NIHSS) score of 0, as isolated visual field deficits may be underrepresented in the NIHSS despite clinically significant posterior circulation stroke. Non-contrast computed tomography of the brain (**A**) demonstrated hypoattenuation in the right occipital lobe (yellow arrow), consistent with a posterior circulation ischemic stroke (PCIS). PCIS accounts for approximately 20% of ischemic strokes and is frequently under-recognized due to non-specific clinical presentations and the relative insensitivity of the NIHSS to posterior circulation events [1,2,3]. Computed tomography angiography of the head and neck demonstrated occlusion of the terminal P4 branch of the right posterior cerebral artery, in the absence of vertebral artery dissection or large-vessel atherosclerosis, supporting a thromboembolic mechanism [4,5]. Magnetic resonance imaging of the brain confirmed the diagnosis, with diffusion-weighted imaging (**B**) demonstrating a hyperintense lesion with corresponding hypointensity on the apparent diffusion coefficient sequence, and fluid-attenuated inversion recovery (FLAIR) imaging (**C**) showing concordant hyperintensity in the right occipital lobe. Given the embolic pattern and posterior circulation involvement, the patient was admitted to the stroke unit for further investigation. Initial management included aspirin (100 mg daily) and high-intensity statin therapy with atorvastatin (80 mg daily). During inpatient admission, a thrombophilia screen was performed to evaluate for potential prothrombotic conditions. This included testing for lupus anticoagulant, Protein C, free Protein S, Factor V Leiden mutation, prothrombin gene mutation, and antithrombin III levels. All results were within normal limits.

**Figure 2:**
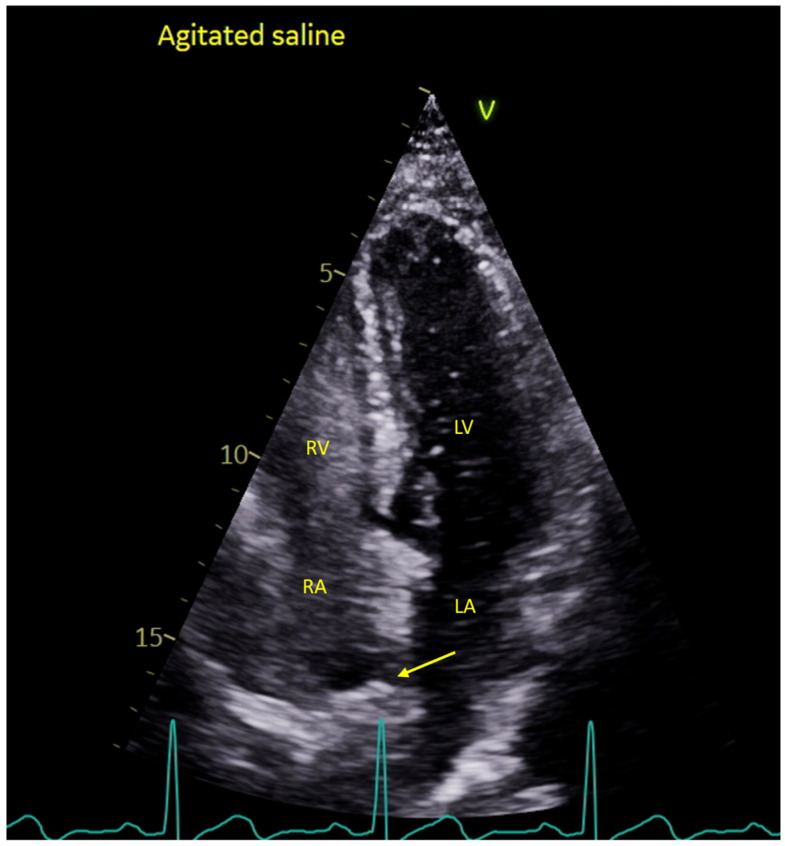
**Transthoracic echocardiography with agitated saline demonstrating an intracardiac shunt and passage of bubbles from right-to-left (yellow arrow).** Transthoracic echocardiography (TTE) was performed to evaluate the presence of an intracardiac shunt using agitated saline contrast (“bubble study”). Echogenic microbubbles do not traverse the pulmonary circulation; therefore, their appearance in the left-sided cardiac chambers indicates a right-to-left shunt [6]. Intracardiac shunts represent a potential mechanism for paradoxical embolism, whereby thrombotic material originating in the venous circulation gains access to the systemic arterial circulation, including the posterior cerebral circulation [7]. In this case, her TTE demonstrated normal left and right ventricular size and systolic function. There was early right-to-left passage of agitated saline on the apical four-chamber view within three cardiac cycles (Figure 2; Video S1), consistent with the presence of an intracardiac shunt. Formal screening for venous thrombosis, including lower limb Doppler ultrasound or D-dimer testing, was not performed during the admission. Nevertheless, the postoperative state following recent surgery was considered a potential prothrombotic condition. The differential diagnosis for right-to-left shunting identified by contrast echocardiography includes patent foramen ovale (PFO) as the most common cause, followed by atrial septal defects and, less frequently, pulmonary arteriovenous malformations [8]. Although there is no formal guideline consensus regarding the sensitivity and specificity of TTE with agitated saline, it is widely recommended as the preferred initial screening modality over two-dimensional transthoracic echocardiography with color Doppler alone for the detection of right-to-left shunts [6]. Assessment of thrombosis risk factors is important in patients with an intracardiac shunt, as such defects may permit embolic material originating in the venous circulation to enter the systemic arterial circulation while bypassing pulmonary filtration. In this patient, several relevant prothrombotic risk factors were present, including obesity (body weight 107 kg), heavy tobacco use (approximately 40 cigarettes per day), and a history of hypertension. However, she had no prior history of venous thromboembolism or cancer. LA = left atrium; LV = left ventricle; RA = right atrium; RV = right ventricle.

**Figure 3:**
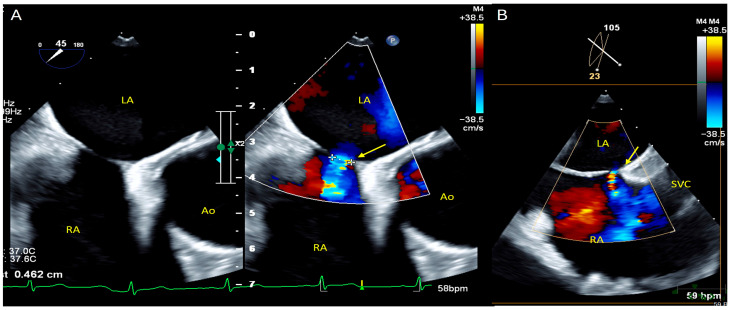
**Transesophageal echocardiography confirming an ostium secundum atrial septal defect.** Transesophageal echocardiography (TEE) was subsequently performed to confirm and characterize the anatomical nature of the intracardiac shunt. While transthoracic echocardiography serves as an effective initial screening tool, TEE is considered the gold-standard imaging modality for the definitive identification and characterization of interatrial communications, including patent foramen ovale and atrial septal defects [6,8,9]. The examination was performed using an X8-2t transesophageal probe (xMatrix TEE, Philips Medical Systems, Andover, MA, USA). Transesophageal echocardiography in the short-axis view (**A**) demonstrated an ostium secundum atrial septal defect measuring approximately 4.6 mm, shown on grayscale imaging ((**A**), **left panel**) and with bidirectional flow on color Doppler ((**A**), **right panel**). The defect was further visualized measuring approximately 4.6 mm on transesophageal echocardiography in the bicaval view (**B**), clearly delineating the atrial septal defect (yellow arrow). Isolated atrial septal defects, as observed in this case, are classified as simple congenital heart defects in which a deficiency of the atrial septum allows abnormal interatrial communication. They account for approximately 6–10% of all congenital heart defects and represent the most common acyanotic congenital cardiac lesion [10]. The estimated prevalence of atrial septal defects in adults is approximately 0.88 per 1000 individuals [11]. Atrial septal defects are classified into four anatomical subtypes: ostium secundum, ostium primum, sinus venosus, and coronary sinus defects. Ostium secundum defects constitute approximately 80% of all atrial septal defects and represent the only true deficiency of the fossa ovalis region, without involvement of adjacent cardiac structures or atrioventricular valves [12]. Ao = aorta; LA = left atrium; RA = right atrium; SVC = superior vena cava.

**Figure 4:**
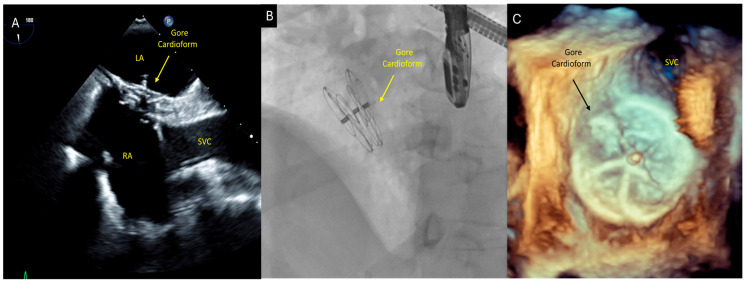
**Percutaneous closure of the atrial septal defect.** Given the presence of an ostium secundum ASD with right-sided chamber enlargement and the occurrence of a presumed embolic stroke without an alternative identified source, percutaneous closure was recommended following multidisciplinary discussion. The patient subsequently underwent successful percutaneous closure of the atrial septal defect using a 30 mm Gore Cardioform septal occluder device, achieving complete shunt resolution. TEE in the bicaval view (**A**) demonstrates the 30 mm Gore Cardioform septal occluder (W. L. Gore & Associates, Inc., Flagstaff, AZ, USA) well seated across the interatrial septum with stable positioning. A fluoroscopic image obtained immediately post-deployment (**B**) confirms correct device expansion and positioning. A three-dimensional en-face transesophageal echocardiographic view (**C**) provides detailed visualization of the occluder device in situ, confirming appropriate coverage of the defect and device stability. The patient recovered well with no recurrent neurological symptoms following ASD closure. Follow-up echocardiography demonstrated stable device position with no residual interatrial shunt. This case suggests presumed paradoxical embolism through an unrecognized atrial septal defect as a possible mechanism of postoperative stroke, after the exclusion of other identifiable causes. The diagnostic sequence—beginning with targeted neuroimaging, followed by contrast-enhanced transthoracic echocardiography and definitive transesophageal assessment—highlights the essential role of multimodality imaging in identifying occult interatrial communications in adult patients presenting with embolic stroke of undetermined origin. LA = left atrium; RA = right atrium; SVC = superior vena cava.

## Data Availability

No new data were created or analyzed in this study.

## Supplementary Materials

The following supporting information can be downloaded at: https://www.mdpi.com/article/10.3390/diagnostics16060907/s1.

## Abbreviations

The following abbreviations are used in this manuscript:

ADC	Apparent diffusion coefficient
Ao	Aorta
ASD	Atrial septal defect
CT	Computed tomography
DWI	Diffusion-weighted imaging
FLAIR	Fluid-attenuated inversion recovery
LA	Left atrium
LV	Left ventricle
MRI	Magnetic resonance imaging
RA	Right atrium
RV	Right ventricle
SVC	Superior vena cava
SWI	Susceptibility weighted imaging
TEE	Transesophageal echocardiography

## References

[1] Alemseged F., Rocco A., Arba F., Schwabova J.P., Wu T., Cavicchia L., Ng F., Ng J.L., Zhao H., Williams C. (2022). Posterior National Institutes of Health Stroke Scale improves prognostic accuracy in posterior circulation stroke. Stroke.

[2] Go S. (2015). Posterior circulation ischemic stroke. Mo. Med..

[3] Grewal K., Austin P.C., Kapral M.K., Lu H., Atzema C.L. (2015). Missed strokes using computed tomography imaging in patients with vertigo: A population-based cohort study. Stroke.

[4] Imam Y.Z., Chandra P., Singh R., Hakeem I., Al Sirhan S., Kotob M., Akhtar N., Kamran S., Al Jerdi S., Muhammad A. (2024). Incidence, clinical features, and outcomes of posterior circulation ischemic stroke: Insights from a large multiethnic stroke database. Front. Neurol..

[5] Caplan L.R., Wityk R.J., Glass T.A., Tapia J., Pazdera L., Chang H.M., Teal P., Dashe J.F., Chaves C.J., Breen J.C. (2004). New England Medical Center posterior circulation registry. Ann. Neurol..

[6] Bernard S., Churchill T.W., Namasivayam M., Bertrand P.B. (2021). Agitated saline contrast echocardiography in the identification of intra- and extracardiac shunts: Connecting the dots. J. Am. Soc. Echocardiogr..

[7] Lin J., Carraro do Nascimento V., Hefford J., Vo T., Matta M.G. (2025). Coronary embolism from a sinus venosus atrial septal defect with partial anomalous pulmonary venous drainage. JACC Case Rep..

[8] Tighe D.A., Aurigemma G.P. (2010). Right-to-left shunts and saline contrast echocardiography. Chest.

[9] Khan R., Karim M.N., Hosseini F., Fine N. (2022). Diagnostic accuracy of transthoracic echocardiography with contrast for detection of right-to-left shunt: A systematic review and meta-analysis. Can. J. Cardiol..

[10] Kheiwa A., Hari P., Madabhushi P., Varadarajan P. (2020). Patent foramen ovale and atrial septal defect. Echocardiography.

[11] Martin S.S., Shapiro E.P., Mukherjee M. (2015). Atrial septal defects: Clinical manifestations, echocardiographic assessment, and intervention. Clin. Med. Insights Cardiol..

[12] Bradley E.A., Zaidi A.N. (2020). Atrial septal defect. Cardiol. Clin..

